# Aging and emotional expressions: is there a positivity bias during dynamic emotion recognition?

**DOI:** 10.3389/fpsyg.2015.01130

**Published:** 2015-08-04

**Authors:** Alberto Di Domenico, Rocco Palumbo, Nicola Mammarella, Beth Fairfield

**Affiliations:** ^1^Department of Psychological Sciences, University of ChietiChieti, Italy; ^2^Schepens Eye Research Institute, Harvard Medical School, BostonMA, USA

**Keywords:** aging, positivity bias, emotion recognition, facial expression recognition, face perception

## Abstract

In this study, we investigated whether age-related differences in emotion regulation priorities influence online dynamic emotional facial discrimination. A group of 40 younger and a group of 40 older adults were invited to recognize a positive or negative expression as soon as the expression slowly emerged and subsequently rate it in terms of intensity. Our findings show that older adults recognized happy expressions faster than angry ones, while the direction of emotional expression does not seem to affect younger adults’ performance. Furthermore, older adults rated both negative and positive emotional faces as more intense compared to younger controls. This study detects age-related differences with a dynamic online paradigm and suggests that different regulation strategies may shape emotional face recognition.

## Introduction

Face perception is one of the most well developed visual skills in human beings. Moreover, it is a skill present from the very early stages of life (Johnson et al., 1991) and holds a crucial role in social communication (Haxby et al., 2000). Indeed, we describe feelings, intentions, motivations, impressions, and above all, emotions based on faces that reveal a large amount of information to the perceiver and at least six emotional expressions expressed by the human species are communicated through facial expressions. In fact, happiness, fear, surprise, anger, disgust, and sadness are typically identified with extreme precision even when shown in static or dynamic images (Howell and Jorgensen, 1970; Buck et al., 1972; Wagner et al., 1986; Ekman et al., 1987). Most importantly, face perception, although sensitive to aging and clinical conditions, plays an adaptive role (Zebrowitz et al., 2015).

Interestingly, contrary to this adaptive function in which we would expect negative faces to have an advantage, literature in emotional face recognition has constantly identified a behavioral recognition advantage for happy faces with respect to negative ones (Calvo and Beltrán, 2013). One of the reasons for this advantage may be that participants in these studies are generally asked to recognize only a final version of an emotional face. Here, we were interested in examining emotional biases and preferences in online recognition of emotional faces that may be more closely related to motivational preferences (Fairfield et al., 2015a).

Increasing evidence shows that face recognition may be impaired in older adults. Studies investigating the effects of aging on face perception using tasks such as face detection (Norton et al., 2009), face identification (Habak et al., 2008; Megreya and Bindemann, 2015) and emotion recognition (Calder et al., 2003) have shown how older adults are slower and less accurate on these face perception tasks (Hildebrandt et al., 2011, 2013). More importantly, aging seems to be related to qualitative changes as well as quantitative changes in face perception [e.g., reaction time (RTs), accuracy etc.]. Different fields of psychology such as perception and memory have shown that older adults seem to show a preference for positive emotional stimuli, a phenomenon referred to in the literature as the positivity effect. This effect on working memory is well documented in literature (Mammarella et al., 2012; Fairfield et al., 2013) and many studies on the effects of aging on memory have highlighted enhanced memory for positive-valence autobiographical events (Kennedy et al., 2004) and in remembering positive images (Mikels et al., 2005) compared to younger adults. In addition, studies on trait impression have shown that older adults tend to judge faces as more positive than younger adults and to perceive faces as more trustworthy as well as less hostile and less dangerous, especially for the most threatening-looking faces (Ruffman et al., 2006; Castle et al., 2012; Zebrowitz et al., 2013).

Traditionally, tasks that assess emotion perception use static facial stimuli representing happy, fear, and neutral expressions but a potentially important factor influencing visual emotion perception concerns the role of dynamic information. It has been reported that healthy controls show an improvement in emotion recognition for dynamic over static point-light displays (Atkinson et al., 2004). Dynamic stimuli therefore present an interesting case for investigating emotion perception in aging. In fact, few studies have assessed the threshold of intensity at which emotions are most consistently identified.

Previous studies have used dynamic emotion recognition tasks based on real videos (Banziger et al., 2009; Minardi, 2012), but here we adopted a new online task. Starting from two pictures of the “Karolinska Directed Emotional Faces” (Lundqvist et al., 1998) portraying the same actor, we generated several morphs and subsequently created videos of faces in which facial expressions changed their intensity from neutral to happy or from neutral to angry. In this way, we have been able to examine whether normal aging is associated with reduced perceptual processing of emotional cues and to determine whether older adults require more intense stimuli to correctly label and discriminate emotional facial expressions. We recorded RTs in facial expression recognition in younger and older adults. In line with facial expression recognition literature, we expected older adults to perform more slowly than younger adults. In addition, to investigate the direction of emotions (i.e., positivity bias for older adults), we asked participants to rate angry, negative and hybrids faces on a visual analogic scale from positive to negative. In this case, we predicted that older adults would rate faces more positively than younger ones.

## Materials and Methods

### Participants

A group of 40 younger and 40 healthy older adults who scored high on the Mini-Mental State Examination (MMSE; Folstein et al., 1975; *M* = 28.75, SD = 1.1; maximum score = 30) participated in the experiment after giving written informed consent in accordance with the the Declaration of Helsinki. The study was approved by the local departmental ethical committee. Participants’ demographic and clinical characteristics are presented in **Table 1**. Exclusion criteria included history of severe head trauma, stroke, neurological disease, severe medical illness or alcohol or substance abuse in the past 6 months. All participants reported normal or corrected-to-normal visual and auditory acuity and younger and older adults reported being in good health.

**Table 1:** Participants’ demographic characteristics.

	Older adults	Younger adults
N	40	40
Age	70.25 (7.2)	23.63 (3.9)^∗^
Gender (% female)	52.5	50
Education (years)	11.43 (4.3)	12.1 (3.9)
MMSE	28.75 (1.1)	

*Values are means (SD) or as otherwise indicated. ^∗^*p* 0.001.*

### Stimuli

We created 20 dynamic videos from two versions of the same actor selected from the “Karolinska Directed Emotional Faces” (Lundqvist et al., 1998). The first version was neutral while the second was happy or angry (gender of the actors and emotions were balanced across trials). These two pictures were then morphed to obtain 98 hybrid faces with an increasing percentage of happiness or anger and these 100 pictures were presented, from the neutral to the happy/angry, for 40 ms in order to generate the video.

### Procedure

#### Recognition Phase

The recognition phase was split into two identical sessions to avoid fatigue. In each session, participants watched 10 videos in the center of the screen and then complete a forced choice recognition test.

During the videos, an initially neutral face gradually changed to assume an expression of happiness or anger. Each video, preceded by a 200 ms fixation point, lasted 4000 ms. Participants pressed the space bar as soon as they were able to identify the emotional expression the face was assuming. Participants subsequently pressed the “m” key if the face had assumed a positive expression or the “z” key if the face had assumed a negative one.

#### Rating Phase

**FIGURE 1:**
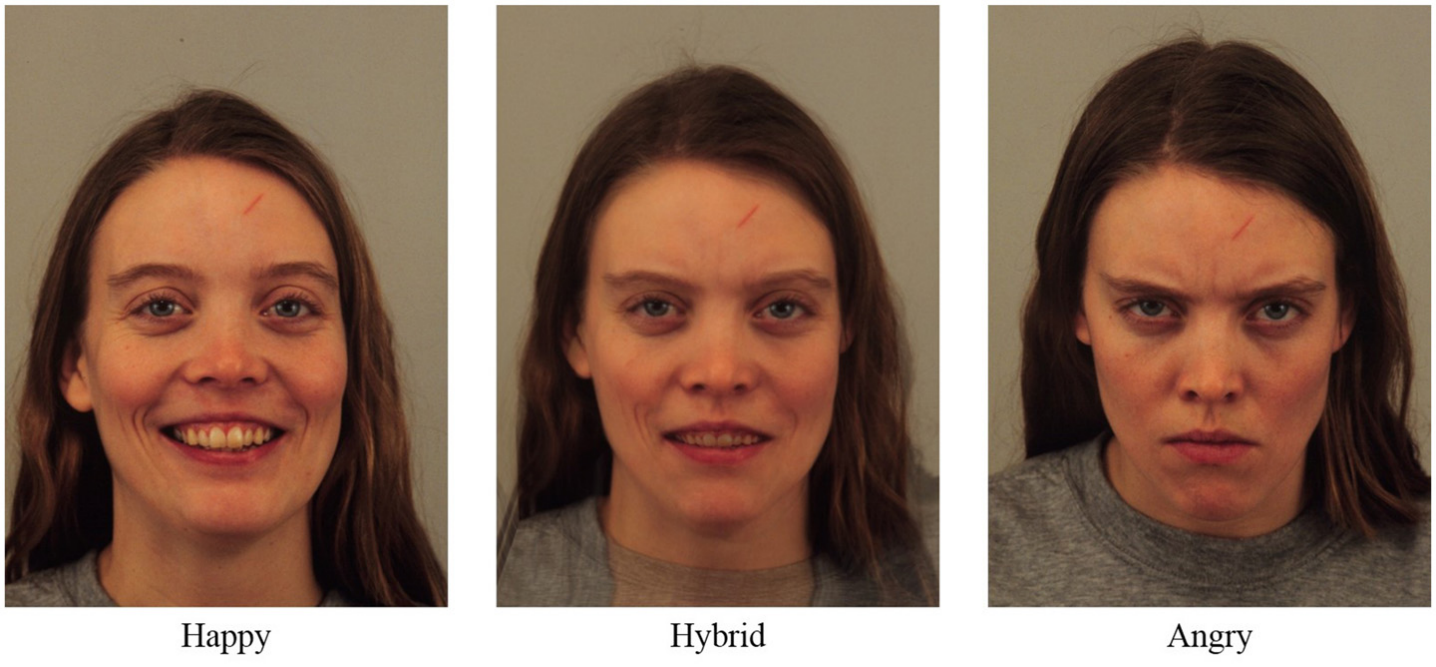
**Example of hybrid face**.

Participants rated 24 new faces according to valence. Six faces were happy, six faces were angry and 12 faces were hybrid (**Figure 1**). Each hybrid face (50% happy and 50% angry) was created starting from two pictures of the “Karolinska Directed Emotional Faces” portraying the same actor; the first picture was happy and the second was angry (gender of actors was balanced across trials). Each face, preceded by a 200 ms fixation point, was presented in the center of the screen for 1000 ms. Participants were then instructed to evaluate, using a visual analog scale (i.e., a line presented horizontally in the center of the screen), how positive or negative the face seemed by moving a slider along the line with the mouse. The line represented a double-ended continuum where the two ends indicated the maximum value of positivity on one side and the maximum value of negativity on the opposite side. The direction of the continuum positive/negative or negative/positive was balanced across participants.

## Results

First, the *t*-test on recognition accuracy did not show any significant differences between groups (*t* = 1.71, *p* = 0.09). This indicates that older and younger adults were equally able to process, label and discriminate faces.

Second, we submitted the accuracy scores (percentage) for facial expression changes to a 2 (*Emotion*: Happy, Angry) × 2 (*Group*: Younger vs. Older Adults) mixed-design analysis of variance. No significant effect were found, indicating that there were no differences in discriminating the changing to happy versus changing to angry for both groups, younger and older adults (the average accuracy was 96%).

**FIGURE 2:**
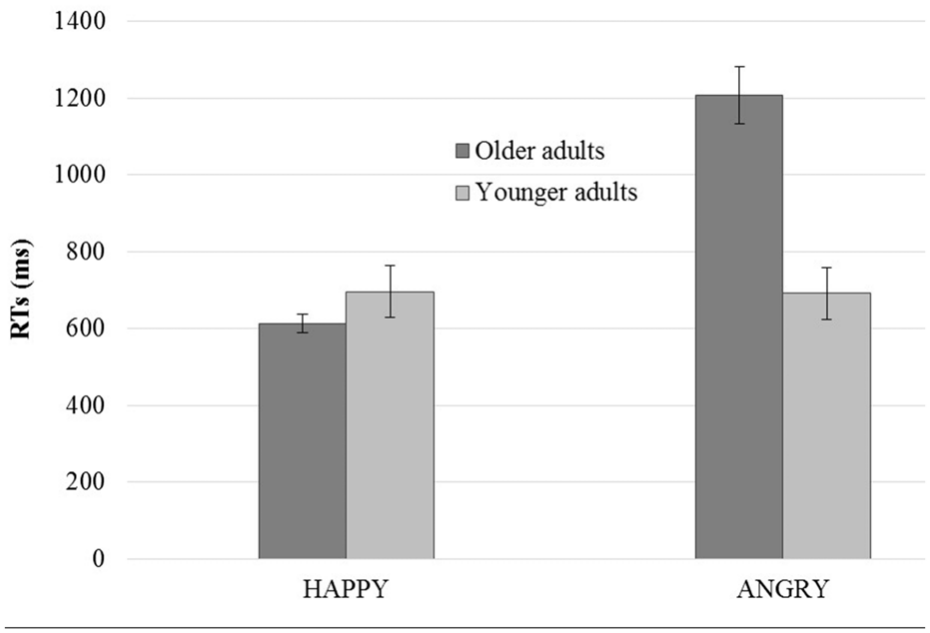
**Reaction times (RTs) for facial expression changes**.

Third, in order to evaluate differences between groups in the temporal processing of the facial expression changes, we submitted RTs to a 2 (*Emotion*: Happy, Angry) × 2 (*Group*: Younger vs. Older Adults) mixed-design analysis of variance. The mixed ANOVA revealed a main effect of group (*F*_1,78_ = 9.99, *p* < 0.01) since younger adults were faster than older adults, a main effect of emotion (*F*_1,78_ = 30.08, *p* < 0.001) because participants recognized changes from neutral to happy faster than changes to angry and a significant two way *Emotion* × *Group* interaction (*F*_1,78_ = 31.19, *p* < 0.001). The *post hoc* analysis on the *Emotion* × *Group* interaction confirmed that older adults were slower to recognize changes from neutral to angry (*M* = 1208.5) compared to happy (*M* = 613.1, *p* < 0.001). No differences were found in the RTs of younger adults (*p* = 0.94; **Figure 2**).

**FIGURE 3:**
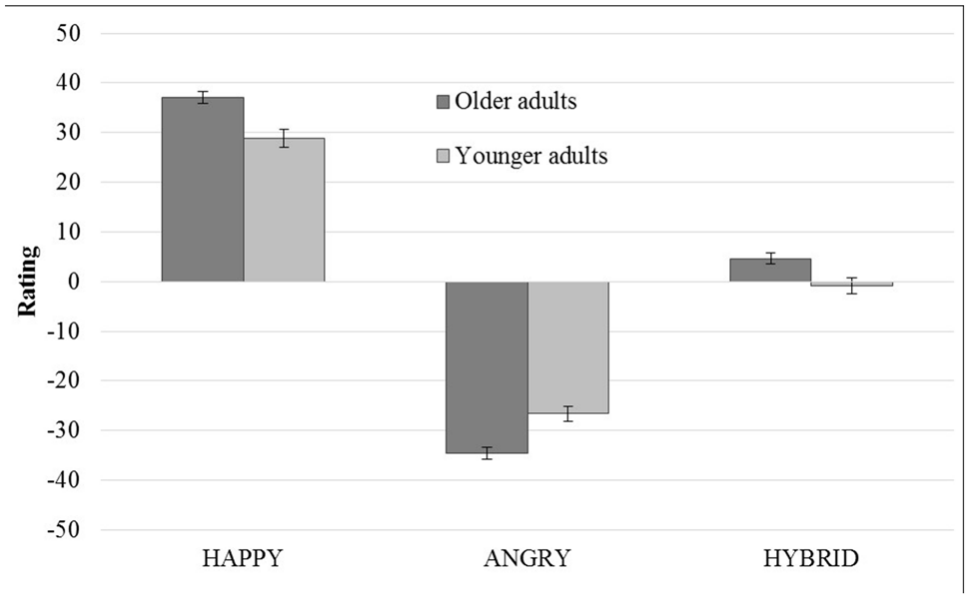
**Facial expressions ratings**.

Finally, in order to examine differences between groups in facial expression ratings, we submitted the face judgment ratings to a 3 (*Emotion*: Happy, Hybrid, Angry) × 2 (*Group*: Younger vs. Older Adults) mixed-design analysis of variance. The mixed ANOVA revealed a main effect of group (*F*_1,78_ = 4.72, *p* < 0.05) because younger adults judge faces more negatively than older adults, a main effect of emotion (*F*_1,78_ = 838.74, *p* < 0.001) and a significant two-way *Emotion* × *Group* interaction (*F*_2,156_ = 15.59, *p* < 0.001). The *post hoc* analysis confirmed that older adults rated negative facial expressions more negatively (*M* = -34.53) than younger adults (*M* = -26.64) and positive facial expressions more positively (*M* = 37.09) than younger adults (*M* = 28.77). Older adults also rated the hybrid faces as more positive (*M* = 4.65) than younger adults (*M* = -0.85; **Figure 3**).

## Discussion

The aim of this paper was to examine what aspects of emotional facial recognition are impaired in older adults by using a novel emotional face recognition task that combines a dynamic recognition phase with a more general static facial rating. Accuracy data indicated that both groups are able to perform the task correctly. However, when we analyzed RTs, we found that older and younger adults showed different patterns of recognition based on face expression. Older adults detected happy expressions faster than angry expressions while younger adults did not show any differences in the time it took them to recognize facial expression. This pattern of performance seems to be linked to the emotional valence of the facial expression since we did not find any differences between the two groups when we asked them to complete a subsequent forced choice recognition phase to evaluate general recognition difficulties. All together, these results seem to suggest a positivity bias during dynamic emotion recognition in older adults. We did not find a happy face advantage typically found in younger adults. This may be because participants did not recognize a single final face in our study, but pressed a key as soon as they were able to detect the direction of the emotional change on a face. The recognition task in itself was very easy and led to ceiling effects in the younger adults that may have “hidden” the happy face advantage. In addition, we found that older adults evaluate unambiguous emotional faces of both valences more intensely than controls. Interestingly, when faces are ambiguous, as in the hybrid condition, only the older adults maintain more intense ratings for positive faces compared to younger adults.

Older adults exhibited enhanced recognition of happy expressions. This finding is consistent with literature showing that older adults prefer positive emotional stimuli (Mather and Carstensen, 2005; Mammarella et al., 2013; Di Domenico et al., 2014). It is possible that age-related motivational changes guide the processing of emotional information and subsequently lead to emotional effects. In fact, older adults often show enhanced memory for positive emotional information. Accordingly, they tend to focus less on negative information linked to perceived time limitations that lead to motivational shifts and direct attention to emotionally meaningful goals (Carstensen, 1995; Fairfield et al., 2015b). Differently, younger adults typically perceive time as more expansive and consequently prioritize goals related to knowledge acquisition and are typically motivated toward knowledge-related goals.

However, our results might also be influenced by the fact that older adults favor different facial features (e.g., Wong et al., 2005). Indeed specific parts of the face can drive emotional processing. For example, the mouth for happiness and the eyes for anger (e.g., Schyns et al., 2007). In future studies, may want to investigate the scanning path of older adults compared to younger adults by manipulating experimental emotional faces.

## Conclusion

In our study, the age-related differences in emotional facial expression recognition evidenced how different regulation strategies shape preferences in emotion processing leading older adults to show a preference for positive information, while younger adults prefer negative information. These findings may have implications for developing new clinical treatments in terms of new emotional facial recognition training programs.

## Conflict of Interest Statement

The authors declare that the research was conducted in the absence of any commercial or financial relationships that could be construed as a potential conflict of interest.
